# Mirizzi syndrome associated with hepatic artery pseudoaneurysm: a case report

**DOI:** 10.1186/1752-1947-2-351

**Published:** 2008-11-17

**Authors:** Oliver Anderson, Radwane Faroug, Brian R Davidson, J Antony Goode

**Affiliations:** 1 Royal Free Hospital & University College School of Medicine, University College London, London, UK

## Abstract

**Introduction:**

This is the first case report of Mirizzi syndrome associated with hepatic artery pseudoaneurysm.

**Case presentation:**

A 54-year-old man presented with painful obstructive jaundice and weight loss. Computed tomography showed a hilar mass in the liver. Following an episode of haemobilia, angiography demonstrated a pseudoaneurysm of a branch of the right hepatic artery that was embolised. At surgery, a gallstone causing Mirizzi type II syndrome was found to be responsible for the biliary obstruction and a necrotic inflammatory mass and haematoma were found to be extending into the liver. The mass was debrided and drained, the obstructing stones removed and the bile duct drained with a t-tube. The patient made a full recovery.

**Conclusion:**

This case highlights another situation where there may be difficulty in differentiating Mirizzi syndrome from biliary tract cancer.

## Introduction

Mirizzi syndrome [1] is often not diagnosed on imaging pre-operatively and is commonly mistaken for gall bladder or bile duct cancer (cholangiocarcinoma) [2-5]. Haemobilia can occur with gall bladder cancer but major haemobilia is more commonly associated with sepsis and inflammation producing a pseudoaneurysm of the hepatic artery [6]. We describe a patient who presented with obstructive jaundice and haemobilia with imaging suggestive of gall bladder cancer and which posed a diagnostic and therapeutic challenge.

## Case presentation

**Figure 1:**
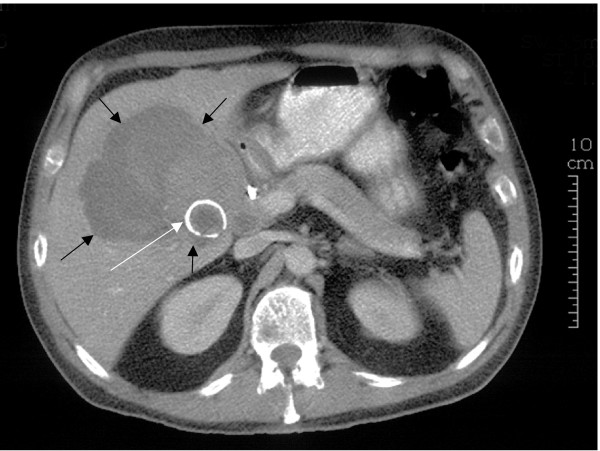
**Abdominal computed tomography scan.** White arrow, ring calcified lesion, gallstone. Black arrows, extent of haematoma.

**Figure 2:**
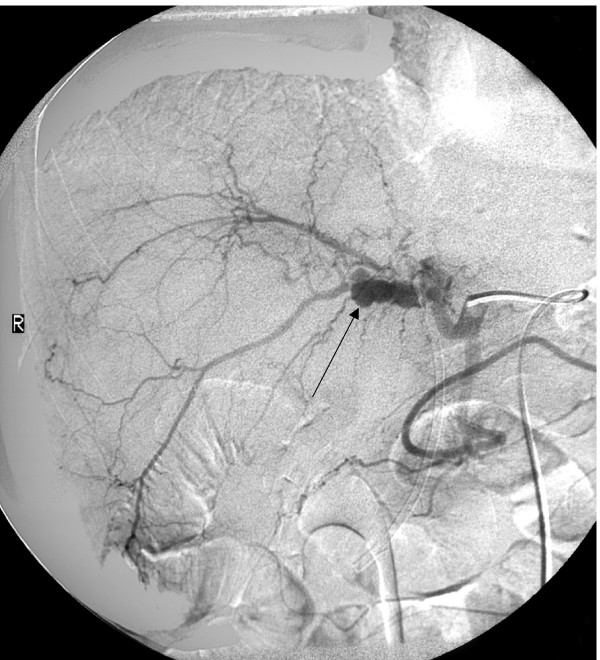
**Hepatic angiogram.** Black arrow, pseudoaneurysm.

A 54-year-old Caucasian man presented with a 4-month history of pain in the right hypochondrium after eating, weight loss and obstructive jaundice. On examination, the patient had a tender mass in the right upper quadrant of his abdomen. Biochemical tests showed changes consistent with obstructive jaundice (bilirubin 76 μmol/litre, alkaline phosphatase 653 U/litre, alanine aminotransferase 281 U/litre, gamma glutamyl transferase (γGT) 1792 U/litre). An abdominal ultrasound scan (USS) showed gallstones in a thick-walled gallbladder and intrahepatic duct dilatation, but no common bile duct (CBD) stones or impacted stone in Hartmann's pouch. Magnetic resonance cholangiopancreatography (MRCP) showed dilatation of the intrahepatic ducts above a common hepatic duct stricture; no gallstone was seen in relation to this. At endoscopic retrograde cholangiopancreatography (ERCP), the hilar stricture was confirmed and stented. Brush cytology was negative for neoplasia. The patient's jaundice resolved. Computed tomography (CT) showed a mass surrounding the gallbladder fossa and a ring-calcified lesion (Figure 1). The mass extended extrahepatically to involve the duodenum and hilar vasculature with encasement of the portal vein and its right and left branches. The appearances were felt to be most in keeping with an advanced gallbladder cancer although the possibility of Mirizzi syndrome was also considered. The patient then passed melaena and became anaemic and required a 4-unit blood transfusion. Oesophagogastroduodenoscopy (OGD) demonstrated bleeding from the ampulla (haemobilia) and no peptic ulcer. Arteriography demonstrated a 3 cm aneurysm of a branch of the right hepatic artery, at the medial aspect of the mass shown on CT (Figure 2). The aneurysm was successfully embolised. Two CT guided percutaneous biopsies of the mass were taken and showed no evidence of neoplasia with only acute inflammatory tissue and necrosis. Due to the doubt over the presence of a malignant process, an exploratory laparotomy was performed. The necrotic hepatic mass was entered and a large volume of organised thrombus and stones was drained from the right lobe of the liver. The gallbladder was excised. A 4 cm gallstone was found to have fistulated from the cystic duct into the common hepatic duct resulting in a Mirizzi type II syndrome. The pseudoaneurysm was immediately superior to the stone. The common bile duct was explored. There were no CBD stones or intrinsic lesion. A t-tube was inserted and a drain was left in the liver cavity. The patient made a full and uneventful recovery and the t-tube was removed after 2 weeks. Histology identified a necrotic inflammatory mass with no neoplasia.

## Discussion

Mirizzi type II syndrome is caused by a gallstone impacting in the neck of the gallbladder eroding the wall of the cystic duct and common hepatic duct and forming a bilio-biliary fistula and obstruction to biliary drainage [7]. Differentiating Mirizzi syndrome from gallbladder cancer or cholangiocarcinoma is a well-recognised problem and it may be impossible to establish the diagnosis pre-operatively [2-5].

Haemobilia is most commonly caused by iatrogenic trauma such as percutaneous biopsy (28%). Aneurysms account for about 10% of cases. Haemobilia can occur secondary to gallstones or neoplasia, and angiography is the most useful diagnostic modality for haemobilia [6].

Aneurysms of branches of the hepatic arteries are rare (0.03% in surgical admissions). At risk groups include patients following orthotopic liver transplant, abdominal trauma, pancreatic pseudocysts, polyarteritis nodosa and percutaneous liver biopsy [8].

In this patient, the haemobilia occurred before the percutaneous biopsies were taken. The presence of haemobilia and hepatic artery aneurysm suggested a benign rather than malignant process. Mirizzi syndrome is likely to have produced cholecystitis, gallbladder perforation and aneurysm formation secondary to sepsis and inflammation.

## Conclusion

No case of Mirizzi syndrome has been reported with a hepatic artery pseudoaneurysm. Embolisation of the aneurysm and open cholecystectomy resulted in a good outcome for this patient. Even when symptoms, signs and radiological appearances are indicative of cancer, benign conditions should be considered when histology is unable to confirm the diagnosis.

## Consent

Written informed consent was obtained from the patient for publication of this case report and any accompanying images. A copy of the written consent is available for review by the Editor-in-Chief of this journal.

## Competing interests

The authors declare that they have no competing interests.

## Authors' contributions

OA prepared the manuscript and performed the literature review. RF prepared the case materials and contributed to the manuscript. BD provided expert opinion and reviewed and corrected the manuscript. AG provided expert opinion and reviewed the radiology.
